# Tumeur stromale du mésentère: à propos d'un cas rare et revue de la littérature

**DOI:** 10.11604/pamj.2015.21.306.6754

**Published:** 2015-08-27

**Authors:** Mamadou Seck, Ibrahima Ka, Mamadou Cissé, Alpha Oumar Touré, Ousmane Thiam, Mohamadou Lamine Gueye, Madieng Dieng, Cheikh Tidiane Touré

**Affiliations:** 1 Service de Chirurgie Générale, CHU Aristide Le Dantec, Dakar, Sénégal

**Keywords:** Tumeur stromale, mésentère, immunohistochimie, chirurgie, imatinib, stromal tumor, mesentery, immunohistochemistry, surgery, imatinib

## Abstract

Les tumeurs stromales du mésentère sont des sarcomes rares du tube digestif. Nous rapportons un cas rare de tumeur stromale dans sa localisation mésentérique. Il s'agit d'un patient admis aux urgences pour abdomen aigu. La tomodensitométrie a objectivé un kyste abcédé du mésentère. L'exploration chirurgicale a retrouvé une tumeur du mésentère. Une exérèse monobloc de la tumeur a été réalisée. L'histologie avec immunohistochimie de la pièce opératoire a mis en évidence une tumeur stromale à risque intermédiaire de malignité. Un traitement adjuvant à base d'imatinib a été ensuite instauré. L’évolution a été simple, sans récidive, avec un recul de 8 mois. Au plan pronostique, selon les critères de Fletcher et de l'AFIP (Armed Forces Institute of Pathology), la tumeur était classée à un risque élevé de récidive. Les tumeurs stromales du mésentère sont exceptionnelles surtout dans leur présentation clinique d'abdomen aigu. Le diagnostic repose sur l'immunohistochimie et le traitement des formes localisées sur la chirurgie, associée à l'imatinib en traitement adjuvant.

## Introduction

Les tumeurs stromales extradigestives (EGIST) sont des sarcomes exceptionnelles, en particulier au niveau du mésentère, de l’épiploon, du péritoine ou des organes rétro-péritonéaux [1, 2]. Longtemps confondues avec le schwanome, le leiomyome et le léiomyosarcome, les progrès de l'immunohistochimie ont permis leur identification par la découverte de deux marqueurs: le CD117 (ou KIT) et le CD34 [3]. Cependant, c'est la découverte effective de mutations génétiques caractéristiques des récepteurs de la famille des tyrosines kinases (C-KIT et PDGFRA) par la biologie moléculaire qui a permis une meilleure approche diagnostique et thérapeutique de cette pathologie [1, 4]. Nous rapportons un cas de tumeur stromale, dans sa localisation mésentérique et sa présentation clinique inhabituelle d'abdomen aigu. L'objectif de cette étude était de discuter les aspects épidémiologiques, diagnostiques, thérapeutiques et pronostiques.

## Patient et observation

### Clinique

Kébé S, 75 ans, hypertendu, sans antécédents personnels ou familiaux, a été admis le 03 Mars 2012 aux urgences chirurgicales du Centre Hospitalier Universitaire Aristide le Dantec de Dakar, pour des douleurs abdominales aiguës, à type piqûre intense, évoluant depuis 48 heures, associées à une constipation chronique, dans un contexte d'altération modérée de l’état général. L'examen général a retrouvé un assez bon état général, une température à 37,7°C, une tension artérielle à 150mmHg/80mmHg et un Pouls à 89p/mn. La taille était de 1,80m, le Poids de 60kg et l'IMC de 18,5. Le patient était symptomatique mais ambulatoire (score OMS à 2). L'examen physique a retrouvé une volumineuse masse abdominale para-ombilicale gauche ferme, mobile et très sensible à la palpation avec une peau en regard normale. Le reste de l'examen était sans particularités.

### Biologie

L'hémogramme a montré un taux de globules blancs à 8200/mm^3^, un taux d'hémoglobine à 8g/dl, un VGM à 84µm3, une CCMH à 30 g/dl et un taux de plaquettes à 748000/mm^3^; La CRP était positive à 192mg/L; la Vitesse de sédimentation était accélérée à 120mm (1^ère^ heure); le bilan rénal était normal avec une créatininémie à 6,17mg/L et une urée à 0,25g/L.

### Tomodensitométrie (TDM)

**Figure 1:**
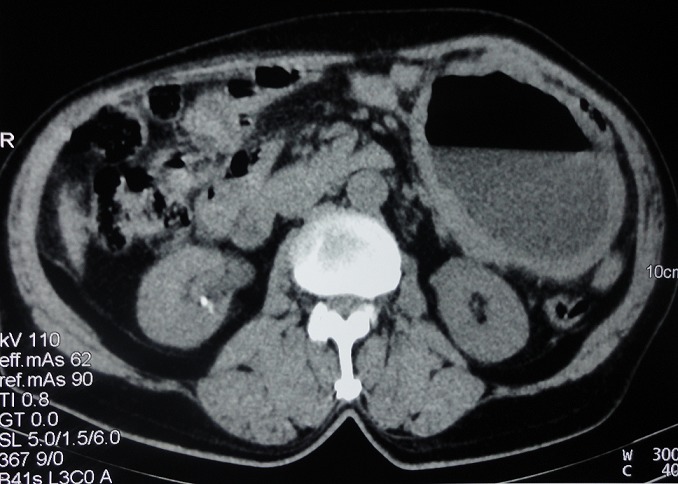
Aspect scannographique de la tumeur

La TDM abdominale a montré une large collection hydro-aérique de 12,5 cm × 10 cm, sous-mésocolique, à paroi épaissie et rehaussée par le contraste, développée aux dépens du mésentère, en faveur d'un kyste abcédé (Figure 1). Il n'y avait pas de localisation secondaire abdominale.

### Prise en charge chirurgicale

**Indication préopératoire**: le diagnostic préopératoire était une tumeur kystique du mésentère, surinfectée par des germes anaérobies (image hydro-aérique dans la masse). La laparotomie exploratrice était alors indiquée. Le délai entre l'arrivée du malade aux urgences et l'intervention était d'environ 6 heures de temps.

**Réanimation préopératoire**: le patient a bénéficié d'une brève réanimation par la mise en place d'une voie veineuse avec remplissage hydro-électrolytique et d'un sondage urinaire.

**Installation et anesthésie**: le patient était installé en décubitus dorsal, sous anesthésie générale avec intubation oro-trachéale et antibioprophylaxie par l'association Amoxicilline-Acide clavulanique et métronidazole.

**Abord**: la voie d'abord a été une laparotomie médiane xypho-pubienne.

**Figure 2:**
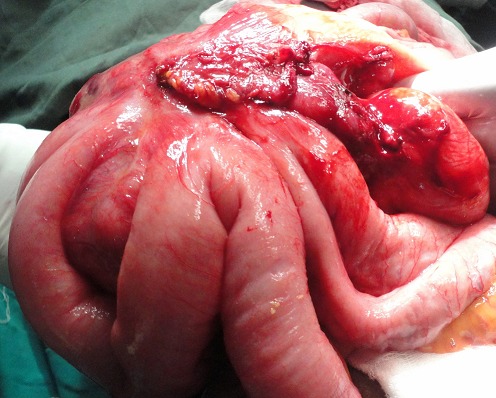
Aspect per-opératoire de la tumeur

**Exploration:** elle a permis de retrouver une volumineuse tumeur du mésentère mesurant 20 cm de grand axe sur laquelle adhéraient 90 cm d'anses grêles iléales et jéjunales (Figure 2). Il n'y avait pas de métastases abdominales macroscopiques.

**Figure 3:**
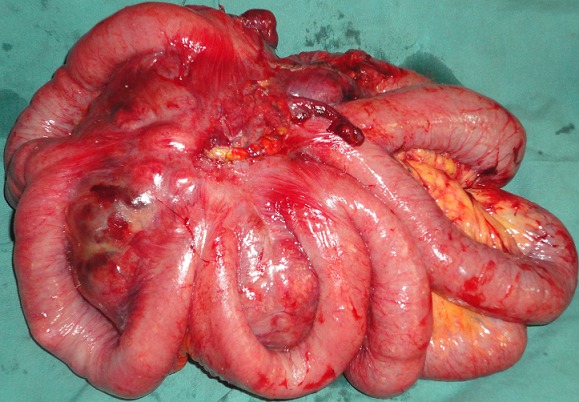
Pièce opératoire

**Gestes**: nous avons procédé à une exérèse complète, sans effraction de la tumeur, emportant 1 m d'anses grêles avec des marges de sécurité de 5 cm (Figure 3). Un rétablissement de la continuité par anastomose jéjuno-iléale termino-terminale a été ensuite réalisée.

### Examen anatomo-pathologique

**Histologie**: l'examen microscopique de la tumeur a montré une prolifération faite de cellules fusiformes présentant des atypies cyto-nucléaires modérées. Les cytoplasmes étaient éosinophiles et les cellules avaient une disposition fasciculée. Il existait d'importantes marques de nécrose. Le contage des mitoses était quasiment impossible. La lésion était développée dans le mésentère et la muqueuse en surface était largement autolysée. Les vingt neuf ganglions prélevés étaient non tumoraux.

**Figure 4:**
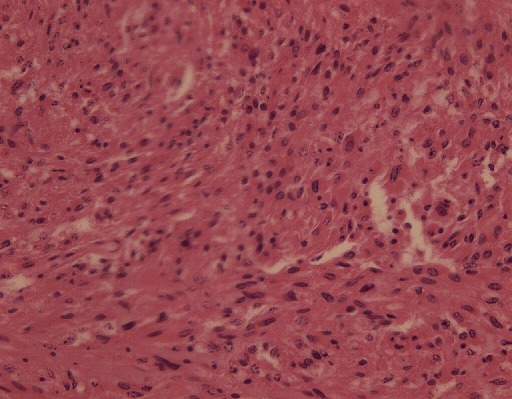
Immunohistochimie

**Immunohistochimie**: seul le marqueur CD117 était positif et l'index de prolifération Ki67 négatif (Figure 4).

### Classification

La tumeur de notre patient était classée à un risque intermédiaire de malignité devant la négativité de l'index de prolifération Ki67, malgré la taille importante et le nombre incomptable de mitoses selon les critères de Yamamoto. Mais, selon les critères de Fletcher et de l'AFIP, la tumeur était classée à un risque élevé de récidive du fait de la taille > 10 cm et du nombre incomptable de mitoses.

### Traitement adjuvant

Notre patient a été mis sous traitement adjuvant à base d'imatinib (GLIVEC^®^) à la dose de 600 mg/j (300mg×2/j) 42 jours après l'intervention.

### Evolution

L’évolution a été simple chez notre patient avec une cicatrisation complète de la plaie cutanée au bout de 15 jours. Le patient a dès lors présenté, avec un recul de 8 mois, un seul effet indésirable au traitement médical (une diarrhée passagère). Nous avons noté globalement une nette amélioration clinique. Le bilan biologique réalisé à 3 mois était normal et la TDM thoraco-abdominale de contrôle réalisée 6 mois après le traitement n'a révélé ni récidive, ni métastases abdominales ou thoraciques.

## Discussion

### Aspects épidémiologiques

L'incidence des tumeurs stromales du mésentère est très faible, car elle représente avec les autres localisations extra-digestives (épiploon et rétropéritoine) moins de 5% [5, 6]. Ces tumeurs surviennent le plus souvent chez l'homme [7–9] L'origine des tumeurs stromales du tube digestif (GIST) a été attribuée aux cellules interstitielles de Cajal (ICC). Par contre, dans les localisations primitives extra-digestives (EGIST) comme le mésentère, l'absence de ces cellules s'oppose à cette théorie. En effet, dans les EGIST, ce sont d'autres types de cellules qui sont incriminés. Il s'agit des cellules ICC-likes qui exprimeraient le CD117 au même titre que les cellules de Cajal [6, 10]. Les facteurs responsables de la mutation des gènes à l'origine des tumeurs stromales sont pour l'instant inconnus [11, 12].

### Aspects diagnostiques

**Aspects cliniques**: dans les tumeurs stromales du mésentère, les manifestations cliniques habituelles sont représentées par des douleurs abdominales et un syndrome de masse abdominale [7, 13]. Par contre, le tableau d'abdomen aigu est rarement décrit dans la littérature [14].

**Aspects radiologiques**: la TDM est actuellement la modalité de choix dans l’évaluation initiale des masses abdominales évocatrices de tumeurs stromales [15]. Dans la littérature, elle a permis de faire le diagnostic présomptif de la plupart des EGIST du mésentère. L'aspect radiologique est le plus souvent une formation kystique avec une hypodensité centrale, un rehaussement périphérique et parfois des calcifications [8, 10, 14]. Dans notre étude, en dehors des calcifications, toutes ces caractéristiques ont été retrouvées. Cependant, le caractère hydro-aérique de la tumeur n'a jamais été rapporté dans la littérature. Cet aspect particulier peut s'expliquer par une surinfection de la nécrose centrale par des germes anaérobies d'origine digestive. Cependant, selon les experts, le bilan d'extension systématique doit comprendre une échographie et une TDM, les autres examens étant à discuter au cas par cas [15].

**Aspects histologiques et immunohistochimiques**: les cellules fusiformes constituent le type histologique le plus fréquent dans les tumeurs stromales [12]. Cependant, dans les formes extra-digestives, le contingent épithélioïde est le plus retrouvé [6, 8, 16]. La fréquence du contingent fusiforme serait liée à une mutation du gène C-KIT et celle du contingent épithélioïde à une mutation du gène PDGFRA [17]. Le grade histopronostique utilisé dans les tumeurs stromales digestives ne semble pas adapté aux EGIST qui ont le plus souvent une taille supérieure à 10cm. En effet, dans une étude récente, les EGIST comme celle du mésentère étaient classées en fonction de l'index mitotique (IM) et de l'index de prolifération Ki67. Pour ces auteurs, les tumeurs ayant un IM ≥ 5cm/CFG et un index Ki67 ≥ 10% seraient de plus mauvais pronostic [18]. D'après ces résultats, la tumeur de notre patient est classée à un risque intermédiaire de malignité, vu la négativité de l'index de prolifération Ki67. Le marqueur immunohistochimique le plus caractéristique des tumeurs stromales est la protéine KIT ou CD117, même si elle est exprimée par d'autres tumeurs telles que le mélanome, le sarcome d'Ewing, l'angiosarcome, le séminome, le carcinome adénoïde kystique et le carcinome bronchique. Il s'agit du marqueur diagnostique le plus spécifique et le plus sensible, car ces tumeurs entre rarement dans le diagnostic différentiel des tumeurs stromales [12].

**Aspects cytogénétiques**: la recherche des mutations génétiques par la biologie moléculaire (BM) a un double intérêt: diagnostique et thérapeutique. Au plan diagnostique, elle est toujours recommandée devant une tumeur stromale à histologie inhabituelle, à immunohistochimie négative, ou lorsque le tableau clinique est très sévère. Les mutations du gène KIT sont surtout localisées sur l'exon 11 et celles du gène PDGFRA sur les exons 18 et 12. La BM permet alors un diagnostic plus précis que l'immunohistochimie [7, 9, 14]. Au plan thérapeutique, la recherche de mutations génétiques permet de mieux adapter le traitement médical. En effet, la présence de mutations de l'exon 11 de KIT est associée à une bonne réponse à l'imatinib, alors que celle de l'exon 9 nécessite de doubler la dose pour une même efficacité.

### Aspects thérapeutiques

**Aspects chirurgicaux**: la voie d'abord chirurgical recommandée est une laparotomie. La résection chirurgicale, macroscopiquement complète avec des marges de résection saines sans curage ganglionnaire, est le meilleur traitement des tumeurs stromales localisées. Le principal risque est l'effraction tumorale qui est associée à un risque élevé de dissémination péritonéale [11]. La résection en bloc est recommandée si possible. La limite optimale des marges de résection n'est toutefois pas bien définie. Le curage ganglionnaire associé n'est pas nécessaire [11]. La nécessité d’établir le diagnostic de ces tumeurs en préopératoire par la biopsie ne fait pas l'objet d'un consensus du fait du risque théorique de l'essaimage de la cavité abdominale (sarcomatose). Elle reste toutefois recommandée par la plupart des experts lorsque la tumeur est localement avancée ou d'emblée métastatique [19].

**Aspects médicamenteux**: dans les tumeurs stromales résécables, le risque évident de récidive après exérèse même complète de la tumeur justifie la mise en route d'un traitement adjuvant par imatinib (risque intermédiaire ou élevé de récidive selon les critères de Yamamoto et al, de Fletcher et de l'AFIP) [3, 18]. Les autres éléments justifiant ce traitement adjuvant sont la localisation extra-gastrique et la nécrose [3, 18]. Le collège américain des chirurgiens oncologistes a montré, dans une étude, que l'imatinib en postopératoire a nettement diminué le taux de récidive et a allongé la survie des patients [20]. Le seul problème que pose l'imatinib est la durée du traitement. Certains auteurs préconisent la poursuite indéfinie du traitement jusqu’à l'apparition d'une progression où d'une toxicité sévère car, une interruption après 1 an de traitement est associée à un haut risque de récidive, même dans les cas de rémission complète [2, 12]. D'autres recommandent un an de traitement car les patients ayant reçu la dose de 400mg/j pendant 1 an ont montré une survie globale de 99% à 1 an, 97% à 2 ans et 97% à 3 ans avec respectivement une survie sans progression de 94%, 73% et 61% [3]. L'imatinib, à la posologie de 400 mg/j, a permis d'obtenir globalement 5% de réponse complète, 45-50% de réponse partielle et 25-30% de stabilité selon les critères RECIST. Ces réponses étaient le plus souvent observées dans les 6 mois suivant l'instauration du traitement mais peuvent survenir plus tardivement. Des résultats similaires ont été rapportés par l’étude de l'US-Finland et celle de l'organisation européenne pour la recherche des cancers, avec une réponse partielle respective de 54% et 53% [2, 12].

**Aspects pronostiques** le pronostic des tumeurs stromales primitives dépend des critères de malignité suivants: la taille, la localisation extra-gastrique, la nécrose, l'index mitotique et l'index de prolifération Ki67. Après résection complète de tumeurs stromales primitives, les récidives sont fréquentes (40 à 80% selon les auteurs). [4, 20]. Le respect des principes d'une chirurgie carcinologique, l'absence d'envahissement ganglionnaire permettent d'améliorer ce pronostic. L'imatinib, en traitement adjuvant améliore d'avantage le pronostic. L'utilisation de l'imatinib, en traitement adjuvant, a augmenté la survie à 1 an de 90% à 98% contre 83% pour les patients sans traitement adjuvant [3, 4]. Mais cette survie dépend surtout du risque de malignité. Elle est proche de la survie de la population générale en cas de risque faible ou intermédiaire et varie entre 0 et 30% en cas de risque élevé [19, 20].

## Conclusion

Les tumeurs stromales du mésentère sont des lésions extrêmement rares, en particulier dans leur présentation d'abdomen aigu. Longtemps confondues avec les autres sarcomes du tube digestif, notamment les léiomyomes et léiomyosarcomes, elles sont aujourd'hui bien individualisées, grâce à l'immunohistochimie. La chirurgie est le traitement curatif des formes localisées. Le pronostic a été amélioré depuis l'introduction de l'imatinib, en particulier comme traitement adjuvant, depuis les dernières recommandations.
